# Incidence and Predictors of Antiretroviral Treatment Modification in HIV-Infected Adults: A Brazilian Historical Cohort from 2001 to 2010

**DOI:** 10.1155/2017/9612653

**Published:** 2017-02-27

**Authors:** Letícia Penna Braga, Cássia Cristina Pinto Mendicino, Edna Afonso Reis, Ricardo Andrade Carmo, Cristiane Menezes de Pádua

**Affiliations:** ^1^Department of Social Pharmacy, Faculty of Pharmacy, Federal University of Minas Gerais, Belo Horizonte, MG, Brazil; ^2^Department of Statistics, Exact Sciences Institute, Federal University of Minas Gerais, Belo Horizonte, MG, Brazil; ^3^Infectious Diseases Reference Center, CTR/DIP Orestes Diniz, Municipal Health Division, Federal University of Minas Gerais, Belo Horizonte, MG, Brazil

## Abstract

This study estimated the incidence of and time to first antiretroviral therapy (ART) modification. This longitudinal analysis comprised a sample of 236 patients from three HIV/AIDS referral centers in Belo Horizonte, Brazil—part of a major historical cohort. Inclusion criteria were as follows: having been treatment-naive patient ≥18 years old who initiated ART between 2001 and 2005 in these three referral centers. The main endpoint was time to first ART modification. Patients were followed up for five years, covering the period 2001–2010, during which time Pearson's chi-square test was performed to compare ART modification between groups. Kaplan-Meier inverse survival curves were employed to describe the probability of ART modification and Cox proportional hazard regression was used to estimate the adjusted hazard ratio (aHR) of ART modification. Among 247 patients from the major cohort, 236 were eligible. Median follow-up time was 37.2 months and the contribution in person-months was 7,615.4 months. A total of 108 (45.8%) patients had their ART regimen modified at least once (incidence rate: 1.42 per 100 person-months). Adverse drug reactions were the main reason for ART modification. Women (aHR = 1.62; *p* = 0.022) and patients on protease inhibitor- (PI-) based regimens (aHR = 2.70; *p* < 0.001) were at higher risk of ART modification.

## 1. Introduction

Antiretroviral treatment (ART) has led to increased life expectancy and has given to HIV infection chronic characteristics that require a new approach to ART use [1–3]. The high number of antiretroviral drug options allows greater possibilities of ART modifications. While this improves treatment possibilities, ART modification may compromise the effectiveness of new treatment regimens due to HIV drug resistance. The risk of therapeutic failure is higher, increasing the need for salvage therapy. Current Brazilian guidelines recommend initiating ART with a nonnucleoside reverse transcriptase inhibitor (NNRTI). Protease inhibitors (PIs) are reserved for second-line treatment, owing to their lower rate of transcriptase reverse enzyme resistance mutations. The disadvantage of this drug class is the higher frequency of adverse reactions compared to NNRTIs. There are many reasons for ART modifications; the most common ones are adverse drug reactions, therapeutic failure, and nonadherence. Therefore, the incidence of and time to first ART modification are important variables to predict the effectiveness of ART [4].

The timing of ART initiation was a point of intensive debate in the last decade. In the early 2000s, Brazilian guidelines recommended initiating ART for those with a CD4+ lymphocyte count below 200 cells/mm^3^ or with clinical manifestations of AIDS. Over the last decade, the recommendations have been modified to start ART earlier, as soon as diagnosed, regardless of CD4+ lymphocyte count or clinical manifestations. In the era of early ART initiation, the need for sustainable antiretroviral regimens will put HIV-infected patients at continuous risk of ART modification [5–7].

Antiretroviral regimen modifications are more likely to occur in the first year of treatment [8]. Most studies have performed this evaluation during this follow-up period. To estimate the incidence of and time to first ART modification in patients receiving lifelong ART, we accessed data from a historical cohort, from 2001 to 2010. The secondary objectives of this study were as follows: describing reasons for ART modifications and comparing the risk of first ART modification based on sociodemographic, clinical, and specific antiretroviral regimens characteristics.

## 2. Methods

### 2.1. Study Design and Population

The present investigation is based on a historical cohort study with the primary objective of evaluating long-term antiretroviral adverse reactions (lipodystrophy, dyslipidemia, and type 2 diabetes) in 247 treatment-naive HIV-infected adults, conducted in three main public HIV/AIDS referral centers in Belo Horizonte, Brazil. These centers are responsible for the care of patients with infectious diseases in Belo Horizonte and its neighboring municipalities. Since 1996, when a national policy of universal access to ART was established, the dispensing of antiretroviral drugs has been performed free of charge and exclusively by public health care centers [9]. Around 11,850 HIV-infected persons received antiretroviral therapy between 2001 and 2013 in the municipal areas and roughly 90% of them were managed by these three centers.

Inclusion criteria were being 18 years or older, being treatment-naïve, and initiating ART between 2001 and 2005 in the three main HIV/AIDS referral centers cited. The inclusion criteria of initiating ART between 2001 and 2005 were adopted based on Brazilian consensus of time to ART initiation. Also, this time frame is suitable for covering important changes that had occurred in the ART guidelines in Brazil as well as worldwide. Participant selection was based on a list of 1,636 treatment-naive infected patients attending the three referral centers and was performed by proportional random sampling, according to the proportion of patients in each center. A total of 297 patients were excluded. The final sample of the main cohort study comprised 247 patients. Further details of the main cohort study have been described and published elsewhere [10].

**Figure 1:**
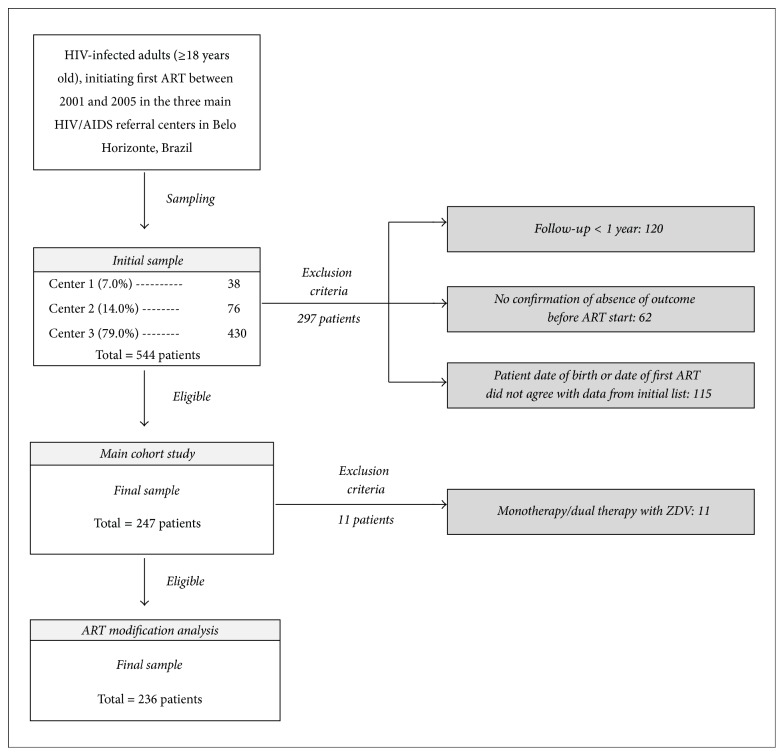
Flowchart of selection of participants from the main cohort study and for the ART modification analysis. ART: antiretroviral therapy. ZDV: zidovudine.

For the study of ART modification, alterations in ART recorded for cohort participants were assessed in follow-up of at least one year and up to a maximum of five years, covering an analysis period from 2001 to 2010. Patients who were using monotherapy or dual therapy with zidovudine (ZDV) were excluded. The final sample included 236 patients (Figure 1). Baseline characteristics regarding sex and age showed no statistically significant differences between eligible and noneligible patients (*p* value = 0.29 and *p* value = 0.25, resp.).

### 2.2. Data Collection and Study Definitions

Data were accessed electronically from medical charts of patients based on an adapted standardized form [11]. Baseline information was collected before and at the time of ART start, and follow-up data were obtained for a maximum of five years thereafter.

The main endpoint was the time to the first ART modification, defined as at least one drug alteration in triple combined ART regimen. Dose adjustment was not considered in the analysis. Time-to-event was defined as the time (in months) from ART start to the date of first ART modification for any reason. The date, reason for modification, and the new regimen prescribed need to be documented in the medical chart. Patients whose treatment was not modified during the follow-up period, deaths, and those lost-to-follow-up patients were censored. Reasons for ART modification were strictly classified as recorded in medical charts, characterized as follows: adverse drug reaction; therapeutic failure (virologic, immunologic, or clinical); nonadherence; and other reasons, which included adverse drug reactions other than those to antiretroviral drugs, unavailability of some antiretroviral at the pharmacy, and drug-interactions. No direct measurement of adherence to treatment was performed.

Predictor variables were sociodemographic characteristics (age at ART start, sex, marital status, city of residence, and occupation category); factors related to health care system utilization (referral center of attendance, mean number of medical visits per year, time between HIV diagnosis and ART initiation, and registration of hospitalization); clinical and laboratory data (T-CD4+ lymphocyte count and viral load at ART start and recording of AIDS-defining illness before first ART prescription); and first ART regimen, classified into two categories: combination of two nucleoside transcriptase inhibitors (NRTIs) and a nonnucleoside transcriptase inhibitor (NNRTI) and two NRTIs and a ritonavir- (RTV-) boosted or unboosted protease inhibitor (PI).

### 2.3. Statistical Analysis

Descriptive analysis was performed by estimating absolute and relative frequencies of variables. Pearson's chi-square tests were performed to compare ART modification between groups. The incidence rate was estimated in person-months, dividing the number of patients who modified ART at least once by the sum of person-time at risk. Kaplan-Meier inverse survival curves were employed to describe the probability of ART modification and the median time to the endpoint. Inverse survival curves in the population categories were compared by the log-rank test. Cox proportional hazard regression model with 95% confidence interval (CI) was used to estimate the hazard ratio (HR) of ART modification. Cox univariate analysis was performed and variables with a *p* value ≤ 0.20 and variables with epidemiologic relevance were included in the multivariate analysis. Shoenfeld's residual analysis was used to test the proportional hazard assumption. For the final multivariate model and the residual analysis, a *p* value ≤ 0.05 was considered. All analyses were performed using Stata software (version 12.0, College Station, USA).

### 2.4. Ethics

The project was approved by Ethical Committee of the Universidade Federal de Minas Gerais (COEP 0017.0.438.203.11) and by participating centers.

## 3. Results

### 3.1. Patients' Characteristics and Antiretroviral Treatment Modification

Among 247 patients from the main nonconcurrent cohort study, 236 were eligible for this analysis. Most of the eligible patients were male and single, divorced, or widowed and resided in the Belo Horizonte municipality. More than a half of the patients (52.5%) were followed up for longer than 36 (mean: 32.7; standard deviation [SD]: 20.6; median: 37.2) months. The mean age at initiation of ART was 37.2 (SD: 9.8) years. Only 17.7% of patients had a mean number of medical visits per year more than four; most (82.6%) attended Center 3, which provides a specialized infectious diseases ambulatory health service. The vast majority of patients (80.4%) had a T-CD4+ lymphocyte count <200 cells/mm^3^ or at least one AIDS-defining illness (CDC/93) at the time of initiating ART.

NNRTI-based regimens were most frequently prescribed (57.2%). Overall, 19 different combinations were prescribed as initial ART, although the combinations zidovudine + lamivudine + efavirenz (ZDV + 3TC + EFV) and zidovudine + lamivudine + nelfinavir (ZDV + 3TC + NFV) accounted for more than 50.0% of all prescriptions (35.2% and 19.9%, resp.). In terms of the NRTI backbone, the combination of ZDV + 3TC was the most frequently prescribed (88.5%), and ZDV was a component of 93.2% of all ART regimens. Didanosine (ddI) was used by 27 (11.4%) patients and stavudine (d4T) by 12 (5.1%). In terms of the NNRTI class, EFV was the most used (68.1%). Among the PIs, nelfinavir (NFV) was the most frequently prescribed (57.4%), followed by RTV-boosted and unboosted indinavir (IDV) (11.9%). The other prescribed PIs were RTV-boosted lopinavir (LPV) (3.0%), RTV-boosted amprenavir (APV) (2.0%), atazanavir (ATV) (1.0%), and saquinavir (SQV) (1.0%).

A total of 108 (45.8%) patients experienced at least one ART modification, corresponding to an incidence rate of 1.42 per 100 person-months. Most patients modified ART once (60.0%), and one patient experienced five ART modifications during the study period. The proportion of first ART modifications was statistically different between patients who started ART with NNRTI-based regimens and those who started with PI-based regimens (Table 1). For 54 (50.0%) patients, the modification occurred during the first year of follow-up, while 30.5% of ART modifications took place between the third and fifth year (Table 2).

**Figure 2:**
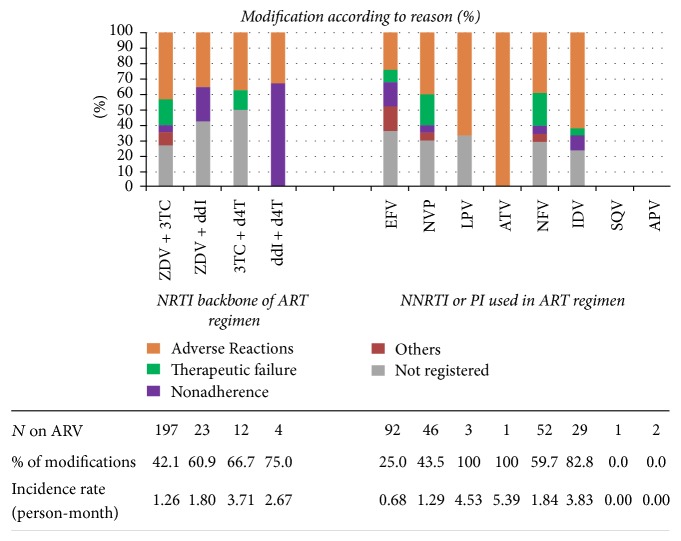
Frequency of ARV modifications according to reason for modification. Values on table shows the total of each combination prescribed, the frequency of modification according to each combination used, and the incidence rate of modifications in person-months. The NRTI backbone is always used combined with one of those NNRTIs or PIs.

Reasons for ART modifications were documented for 77 (71.3%) patients. The main reason was adverse drug reactions (41.7%), followed by therapeutic failure (14.8%) and nonadherence (9.3%). PI-based regimens were the most commonly modified, accounting for 64.3% of all regimens changed. Nelfinavir (NFV), the most prescribed PI, was the antiretroviral drug most frequently switched, accounting for 29.9% of all switches. The main reason was adverse drug reaction. EFV had the lowest incidence rate of modification: 0.68 per 100 person-months (95% CI: 0.52–0.79). In terms of the NRTI backbone, the combination of stavudine (d4T) and didanosine (ddI) was prescribed least frequently but was the most modified, due to nonadherence. Regimens containing saquinavir (SQV) and amprenavir (APV) were the only ones that did not undergo any modification (Figure 2).

A total of 75 (69.4%) patients modified one drug in ART regimen. Another 27 (25.0%) modified two antiretrovirals, and 6 (5.6%) switched all the three drugs in their triple therapy regimen. The combination of ZDV + 3TC + NFV was the most modified of all combinations (30.0%), while the combination of ZDC + 3TC + EFV was the most common ART regimen switched to (21.3%), followed by AZT + 3TC + LPV/r (13.9%). Of a total of 147 substitutions, the most common substitution was from ZDV to d4T (16.3%). EFV was the most common antiretroviral switched to (20.4%), and RTV-boosted LPV was the most common PI switched to, accounting for 18 new ART regimens (41.9% of all newly prescribed PI-based regimens). Among the 18 LPV-based regimens switched to, only 2 patients (11.1%) experienced another ART modification. This rate was 25% for regimens containing ATV and 61.5% for d4T-containing regimens.

### 3.2. Rates and Predictors of First ART Modification

**Figure 3:**
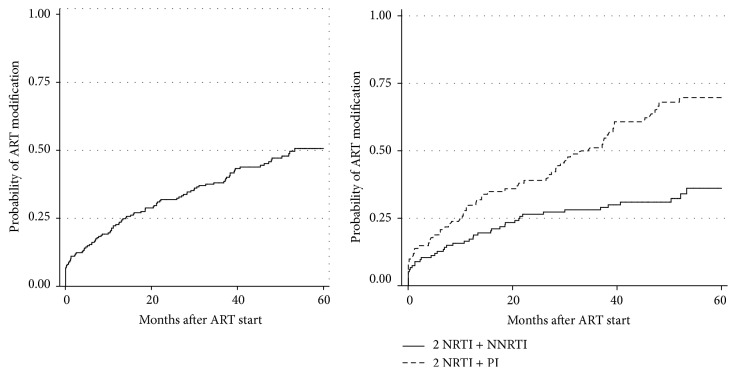
Kaplan-Meier failure estimations of probability of ART modification. On the left: for all 236 patients. On the right: for 236 patients stratified by ART regimen (log-rank test, *p* < 0.001).

The cumulative probabilities of ART modification after 12, 24, 36, 48, and 60 months were 23.2%, 32.6%, 38.7%, 46.7%, and 56.5%, respectively. After 53.3 months of ART, 50% of all patients remained on their initial regimen. Significantly higher modification rates were found for patients who received PI-based regimens compared with those who received NNRTI-based ones (log-rank test, *p* < 0.001) (Figure 3).

The final model in the multivariate analysis included three variables (Table 3). Females were at increased risk of ART modification (aHR = 1.62; 95% CI: 1.07–2.45). Increased risk was found for patients using PI-based regimens (aHR = 2.70; 95% CI: 1.77–4.13). Increased risk of ART modification was also found in patients with AIDS-defining illness in ART beginning, but it was not statistically significant (Table 3).

## 4. Discussion

Our study provides relevant insights into the reasons for ART modification and risk factors associated with modification in historical cohort of HIV/AIDS patients covering data from 2001 to 2010. The sex and age distributions of the study population were similar to those of AIDS populations in Brazil and in the Belo Horizonte municipality reported previously [12, 13]. Other sociodemographic variables related to health care system utilization and clinical characteristics were similar to those described in a cross-sectional study conducted in the same city [14].

During the period 2001–2005, Brazilian national treatment guidelines did not recommend immediate ART initiation at HIV diagnosis. Instead, ART initiation was guided by immunologic and/or clinical criteria, being indicated for patients with a CD4+ T-lymphocyte cell count < 200 cells/mm^3^ or with clinical manifestations of AIDS. During the period 2004-2005, discussions started about initiating ART in asymptomatic patients with higher CD4+ lymphocyte counts [6]. Overall, 68.4% of patients in our study initiated ART with a CD4+ T-lymphocyte count < 200 cells/mm^3^ or clinical manifestations of AIDS, 72.5% of whom initiated ART in 2001 and 2002. This shows that, even with the discussions about when to initiate ART, the initial recommendations were followed, because, during the study period, the evidence was not clear enough to support earlier ART initiation. Despite the criteria used for starting ART, we found a low frequency of hospitalization. The Qualiaids Report, a Brazilian system for evaluating and monitoring the quality of HIV/AIDS healthcare assistance, recommends at least four doctor visits per year for patients starting ART [15]. However, a very low proportion of patients attended an average of four or more doctor visits per year, suggesting that it is important to evaluate barriers to accessing health care.

The incidence of ART modification was higher than that found by other Brazilian and international observational studies [16–19]. This can be explained by different outcome definitions, which can regard discontinuation, modification, or switch to second-line treatment as ART modification. De Castilho et al. (2006) measured the incidence of ART interruption or the switch to another ART and found an ART modification incidence of 35.9% [17]. Even without considering interruption, the incidence of ART modification was higher in our study. This could also be related to our longer follow-up period. Several studies followed patients for up to two years. In our cohort, the number of ART modifications after three, four, or five years of treatment represented 30.5% of the ART modifications, highlighting the importance to study this outcome over for a longer duration of follow-up. Compared with international studies, this difference might also be related to differences in Brazilian HIV/AIDS policies, the availability of ART, and access to HIV/AIDS health care services. A study by Apuzzo et al. (2009) was the only that found a higher incidence of ART modification (56%) than ours [20]. This is most likely due to the bigger sample size, drawn from eight different centers, and the outcome definition taking drug switch, discontinuation, addition, or reduction (discontinuation of one or more drugs) into account.

PIs became available in Brazil in 1996, but until 2004 they were recommended only for patients with clinical manifestations of HIV or with severe immunodeficiency. Since the introduction of triple combined therapy, the NNRTI class has been recommended as first-line treatment, combined with two NRTIs, with EFV being the NNRTI of choice. PIs, combined with two NRTIs, are recommended as alternative antiretroviral regimens. In our sample, EFV-based regimens were the most prescribed overall, and nelfinavir (NFV) was the most prescribed PI. The majority of patients on PI-based regimens presented AIDS-defining illness(es) and initiated ART before 2004. EFV was the most well-tolerated drug, corroborating reports on the good effectiveness and safety profile of this drug, supporting the recommendation that it be the NNRTI of choice [21]. Modern PIs, RTV-boosted LPV and ATV, were the most common PIs switched to and they were well tolerated; few patients on second-line ART regimens with LPV and ATV experienced another ART modification. SQV and APV were the only drugs that were not modified; but they were rarely prescribed. One disadvantage of these drugs is their higher cost compared with standard first-line antiretroviral drugs. This confirms the current recommendation to use RTV-boosted LPV as the PI of choice [22]. It is important to observe that the most common substitution was from ZDV to d4T. Adverse drug reactions associated with ZDV, principally anemia, are widely described, supporting substitution of ZDV [23]. However, more than 60.0% of patients who switched to d4T experienced another ART modification due to adverse drug reactions related to d4T. This strengthens the evidence for the advice, given in 2006, to use d4T with caution and, subsequently, the decision to phase out the use of this drug [24].

Adverse drug reactions were reported to be the main reason for ART modification in most studies, with a higher probability of occurring in the first six months of treatment [8, 18, 20]. Landier et al. (2011), studying the incidence and reasons for switching to second-line ART, found that ART modifications occurred earlier when motivated by adverse drug reactions than by therapeutic failure [19]. This explains the high incidence due to adverse reactions at the beginning of treatment and the result that therapeutic failure was the second most frequent reason for ART modification in our study. Pedrol Clotet et al. (2014) found that treatment simplification was the most frequent reason for ART modification, a reason not described in our cohort [25]. This evaluation was conducted when a larger number of drugs became available and it was possible to have easier drug regimens or dosing, which can improve adherence. We suppose that modifications to more simple therapeutic regimens, with fixed-dose combinations, may be a trend.

Patients who initiated ART with PI-based regimens had a modification rate of 2.70 (CI 95%: 1.77–4.13) compared to those who initiated ART with NNRTI-based regimens. This result is similar to other longitudinal studies [26, 27]. PIs have a lower number of resistance mutations for reverse transcriptase enzymes compared to NNRTIs, which justifies this drug choice in preventing failure. However, PI-based regimens are not as well tolerated as NNRTIs, which explains their higher modification risk [28]. This highlights the need for better clinical evaluation and monitoring in the early phase after initiating PI-based ART. A multicenter Spanish cohort with ARV naïve patients, with viral loads > 100,000 copies/mL, compared EFV to PIs in terms of effectiveness and showed better results with EFV, which also demonstrated better viral load suppression in individuals with very high viral loads (>500,000 copies/mL) and low CD4+ T-cell counts [29]. Considering that the clinical profile of patients in our cohort was similar to that study and given our result of a higher modification rate associated with PIs, initiating ART with this drug class should be carefully evaluated. Even when ART is initiated with a worse clinical condition, initiating ART with NNRTIs could avoid early ART modifications.

In our cohort, women were 1.62 times more likely to have their ART regimen modified than men. This higher rate in women was related to adverse reactions, the most common reason for modification. This result is similar to a university cohort study in the USA which found that women were more likely to discontinue ART due to dermatologic reactions and poor-adherence [30].

Incomplete information in medical records was a limitation of our study. Important demographic and clinical variables, such as individual income and viral load, were not registered appropriately. The registration of reasons for ART modification is required in order to dispense a new antiretroviral drug at the pharmacy. Nevertheless, reasons for 30.6% of the first ART modification were not registered. Viral load was not recorded for all patients, which is an important variable to identify ART failure. This identifies the need for easy, user-friendly registering tools and strategies to promote the incorporation of data logging practices into routine care. Additionally, modifications for treatment simplification were not record and it was not possible to make inferences about that as a reason, which may be linked to adherence. Direct measures of nonadherence or irregularities of ART use could strengthen the association of predictive factors and ART modification.

Despite these limitations, our study supports the high incidence of ART modifications among HIV/AIDS patients. It is innovative because it evaluated ART modification over five years of follow-up. As ART modification is caused by adverse drug reactions, therapeutic failure, and nonadherence, it can be used as a proxy for low treatment effectiveness. Therefore, the monitoring of ART modification becomes crucial to prevent treatment abandonment.

In summary, this historical cohort study showed differences in the risk of ART modification related to sex and ART regimen. Furthermore, it highlighted the importance of evaluating reasons for ART modification in order to support better regimen choices. More sustainable regimens should be accessed, with lower toxicity profiles and simpler dosage requirements to improve adherence. Also, health systems should be prepared to offer health care options to manage complications of HIV treatment and to monitor adverse drug reactions related to ART. It is essential that HIV/AIDS programs have an integrated pharmacovigilance system to improve ART effectiveness and sustainability.

## Figures and Tables

**Table 1:** Baseline characteristics of the HIV/AIDS patients from the historical cohort, Belo Horizonte, Brazil.

Variables	Global (*n* = 236)	ART modification (*n* = 108)	
	*n* ^*∗*^ (%)	*n* ^*∗*^ (%)	*p* value^*∗∗*^
*Sociodemographic*			
Age at ART beginning (years)			0.207
≤35	114 (48.3)	57 (50.0)	
>35	122 (51.7)	51 (41.8)	
Sex			0.461
Male	148 (62.7)	65 (43.9)	
Female	88 (37.3)	43 (48.9)	
Marital status			0.669
Single/divorced/widower	144 (64.9)	64 (44.4)	
Marriage/stable union	78 (35.1)	37 (47.4)	
City of residence			0.737
Belo Horizonte	149 (63.7)	70 (47.0)	
Metropolitan area/countryside	85 (36.3)	38 (44.7)	
Occupation category			0.336
Formal job	155 (70.4)	74 (47.7)	
Unemployment/retired/housemaid	64 (29.2)	26 (40.6)	
*Related to health assistance*			
HIV/AIDS referral center			0.793
Center 1/Center 2	41 (17.4)	18 (43.9)	
Center 3	195 (82.6)	90 (45.2)	
Average medical visits/year			0.433
≤4	191 (82.3)	85 (44.5)	
>4	41 (17.7)	21 (51.2)	
Time between HIV diagnosis and initial ART (days)			0.824
≤90	105 (47.3)	46 (43.8)	
>90	117 (52.7)	53 (45.3)	
Hospitalization			0.440
No	195 (82.6)	87 (44.6)	
Yes	41 (17.4)	21 (51.2)	
*Clinical and laboratorial data*			
Initial CD4+ T-lymphocyte (cells/mm^3^)			0.258
<200	119 (59.2)	59 (49.6)	
200–350	55 (27.4)	23 (41.8)	
>350	27 (13.4)	9 (33.3)	
Viral load (copies/mL)			0.516
≤100.000	105 (65.6)	44 (41.9)	
>100.000	55 (34.4)	26 (47.3)	
AIDS-defining illness			0.145
No	94 (44.3)	36 (38.3)	
Yes	118 (55.7)	57 (48.3)	
First classes of ART regimen prescribed			<0.001
2 NRTI + NNRTI	135 (57.2)	43 (32.6)	
2 NRTI + PI	101 (42.8)	65 (64.3)	
Most commonly first ART combination			<0.001
ZDV + 3TC + EFV	83 (35.2)	20 (24.1)	
ZDV + 3TC + NFV	47 (19.9)	28 (60.0)	
ZDV + 3TC + NVP	37 (15.7)	15 (40.5)	
ZDV + 3TC + IDV	17 (7.2)	10 (58.2)	
ZDV + ddI + NFV	10 (4.2)	7 (70.0)	
ZDV + ddI + NVP	8 (3.4)	4 (50.0)	
ZDV + 3TC + IDV/r	6 (2.5)	5 (83.3)	
d4T + 3TC + EFV	4 (1.7)	2 (50.0)	
d4T + 3TC + NFV	4 (1.7)	2 (50.0)	
Others^*∗∗∗*^	20 (8.5)	15 (75.0)	
Total	236	108	

3TC: lamivudine, ART: antiretroviral therapy, d4T: stavudine, ddI: didanosine, EFV: efavirenz, IDV/r: indinavir/ritonavir, NFV: nelfinavir, NRTI: nucleoside reverse transcriptase inhibitor, and NNRTI: nonnucleoside reverse transcriptase inhibitor. NVP: nevirapine, PI: protease inhibitors, and ZDV: zidovudine.

^*∗*^Missing values were not shown.

^*∗∗*^Pearson's chi-square test.

^*∗∗∗*^Others include ART combinations containing lopinavir, lopinavir/ritonavir, amprenavir, atazanavir, saquinavir, and different combinations of the antiretrovirals presented.

**Table 2:** Frequencies of first ART modification and incidence rate by years after the beginning of treatment.

Year of treatment	First ART modification (%)	% of the 236 patients	Incidence rate (100 person-months)
1st	54 (50.0)	22.9	22.4
2nd	21 (19.4)	8.9	4.0
3rd	13 (12.0)	5.5	1.9
4th	13 (12.0)	5.5	0.8
5th	7 (6.7)	3.0	0.2

*Total*	108 (100)	45.8	1.4

ART: antiretroviral therapy.

**Table 3:** Adjusted hazard ratio and 95% confidence interval (95% CI) estimated by Cox proportional hazards regression in multivariate analysis of the historical cohort for first ART modification, Belo Horizonte, Brazil.

Variables	aHR (95% CI)	*p* value
Sex		
Male	1.00	
Female	1.62 (1.07–2.45)	0.022
AIDS- defining illness		
No	1.00	
Yes	1.49 (0.98–2.27)	0.061
First ART regimen prescribed		
2 NRTI + NNRTI	1.00	
2 NRTI + PI	2.70 (1.77–4.13)	<0.001

aHR: adjusted hazard ratio, ART: antiretroviral therapy, NRTI: nucleoside reverse transcriptase inhibitor, NNRTI: nonnucleoside reverse transcriptase inhibitor, and PI: protease inhibitor.

Multivariate analysis: Shoenfeld's residual test: *χ*^2^= 6.63; d.f. = 3; *p* value = 0.452.
